# Author's Response to Dr. Leo

**DOI:** 10.1371/journal.pmed.0030376

**Published:** 2006-08-29

**Authors:** Patrick Sullivan

**Affiliations:** University of North Carolina, Chapel Hill, North Carolina, United States of America

Jonathan Leo raises issues with the adoption literature on schizophrenia [1]. These studies were intensively and independently scrutinized in the 1980s—see the series of papers by Kendler and Gruenberg (e.g., [2]). Most would agree that the number, size, and quality of adoption studies do not provide the highest-quality data (as discussed at more length elsewhere [3].

However, the salient point in my paper [4] was that this body of work (twin, adoption, and family studies) provides a consistent and solid rationale for the search for genes for schizophrenia.

Dr. Leo's comments about the treatment of schizophrenia are not within the scope of my paper.
